# Successful Treatment of Cutaneous Leishmaniasis With Cryotherapy

**DOI:** 10.7759/cureus.41871

**Published:** 2023-07-14

**Authors:** Lauren A Linquest, Leigh C Hickham, Bayley J Richardson, Patricia R Hickham

**Affiliations:** 1 Dermatology, Louisiana State University Health Shreveport - School of Medicine, Shreveport, USA; 2 Dermatology, Louisiana State University Health Sciences Center New Orleans - School of Medicine, New Orleans, USA; 3 Dermatology, Texas Tech University Health Sciences Center Paul L. Foster School of Medicine, Lubbock, USA; 4 Dermatology, East Jefferson General Hospital, New Orleans, USA

**Keywords:** treatment, efficacy, liquid nitrogen, cryotherapy, cutaneous leishmaniasis, leishmaniasis, clinical dermatology, general dermatology, case report

## Abstract

Leishmaniasis, a protozoal infection, is a growing health concern with 1.5 million new cases reported annually resulting in a wide spectrum of disease and clinical presentations. The disease is endemic in 98 countries with increasing prevalence in non-endemic areas. There are various treatment approaches that are often individualized based on host and parasite factors. Current treatment guidelines and data are variable and provide limited direction for specific treatment plans. Additionally, current recommended therapies are not benign, and are expensive and unavailable to most patients, especially in low-resource areas where leishmaniasis is most prevalent. Here, we report the diagnosis and successful treatment of cutaneous leishmaniasis in a 65-year-old male, who recently traveled to Mexico. Initial treatment with topical antifungals and oral antibiotics was ineffective. After successive treatment with local liquid nitrogen, the lesions completely resolved with no adverse effects or recurrence. Given there is limited evidence-based data supporting cryotherapy treatment as a first-line treatment, this report supports the efficacy of cryotherapy as a safe, cost-effective, and accessible treatment for cutaneous leishmaniasis.

## Introduction

Leishmaniasis is a vector-borne, protozoal infection that affects over 12 million people globally and is endemic in Africa, Asia, the Middle East, as well as Central and South America [1,2,3]. However, international travel, immigration, and abroad military involvement have increased the prevalence of leishmaniasis in developed countries, like the United States [3-6]. Most cases are concentrated in impoverished areas with limited healthcare making it imperative to find an accessible, safe, and inexpensive therapy. The infection is transmitted by a sandfly bite with many patients unaware of the inoculation [1,3]. Clinical presentation ranges from asymptomatic to cutaneous, mucosal, and/or visceral involvement [4]. Disease manifestations are largely dependent on the Leishmania species as well as the stage of the infection [1].

After an average incubation period of two to eight weeks, an erythematous papule emerges at the inoculation site. Over time, the lesion enlarges to form an ulcerated nodule. The ulcer has raised borders with a granulomatous base due to epidermal breakdown [1,4,6]. *Leishmania tropica* typically causes a single ulcer; however, other strains such as *Leishmania major* can result in multiple ulcers [3]. Lesions are generally painless and confined to the face, ears, elbows, and knees but can occur across the entire body [1,6]. Some cutaneous infections disseminate hematogenously, particularly in immunocompromised patients, causing mucosal and visceral leishmaniasis [4,6]. With no treatment, the infection can cause scarring, disfigurement, pulmonary involvement, and death [6].

Shave and punch biopsies are the most common diagnostic tests with sensitivities around 70% [6]. Confirmatory pathology shows intracellular amastigotes in phagocytic cells, preferentially macrophages and dendritic cells, with patterns of granulomatous inflammation [7,8]. A detailed medical history including travel to endemic areas is necessary for diagnosis as pathology has low sensitivity. Risk factors such as a history of HIV infection and other comorbidities should also be noted. Immunocompromised hosts may have more advanced, disseminated presentations of disease due to a diminished adaptive T-cell response [6].

Antileishmanial therapy is largely individualized and determined by the infecting Leishmania species, stage at presentation, extent of disease progression, relative risk between topical, oral, and parenteral medications, and medical judgment [4]. Pentavalent antimonials, such as sodium stibogluconate and meglumine antimoniate, are the first-line therapies for leishmaniasis [6,9]. Distributed by the Centers for Disease Control and Prevention (CDC), these drugs are not widely available and involve repeated painful injections, risk of drug resistance, variable efficacy, and high incidence of adverse effects. Amphotericin B is a second-line therapy that has similar effects as antimonial treatment [1,6]. Other treatments including topical solutions, intralesional injections, intramuscular injections, and thermotherapy have also been used on patients with leishmaniasis with inconsistent cure rates [1]. Given the variability in disease presentation and poor generalizability with current clinical trials, there is limited direction provided by treatment guidelines. Therefore, this case study outlines a specific, clear treatment regimen that was successful in treating cutaneous disease. In this report, we observe how regular cryotherapy treatments alone can treat and cure cutaneous leishmaniasis. 

## Case presentation

**Figure 1:**
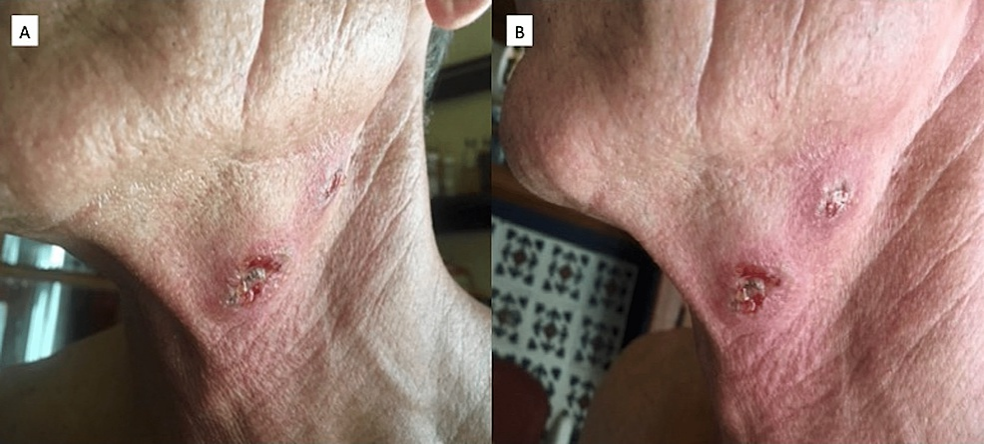
Clinical pictures prior to cryotherapy Image A (Left): Anterolateral view of large anterior neck lesion prior to cryotherapy Image B (Right): Lateral view of two ulcerated, erythematous neck lesions prior to cryotherapy

In early 2018, a 65-year-old male presented with multiple crusty nodules with central depressions as well as erythematous nodules with central ulceration and necrosis located on the left lateral neck as seen in Figure 1.

**Figure 2:**
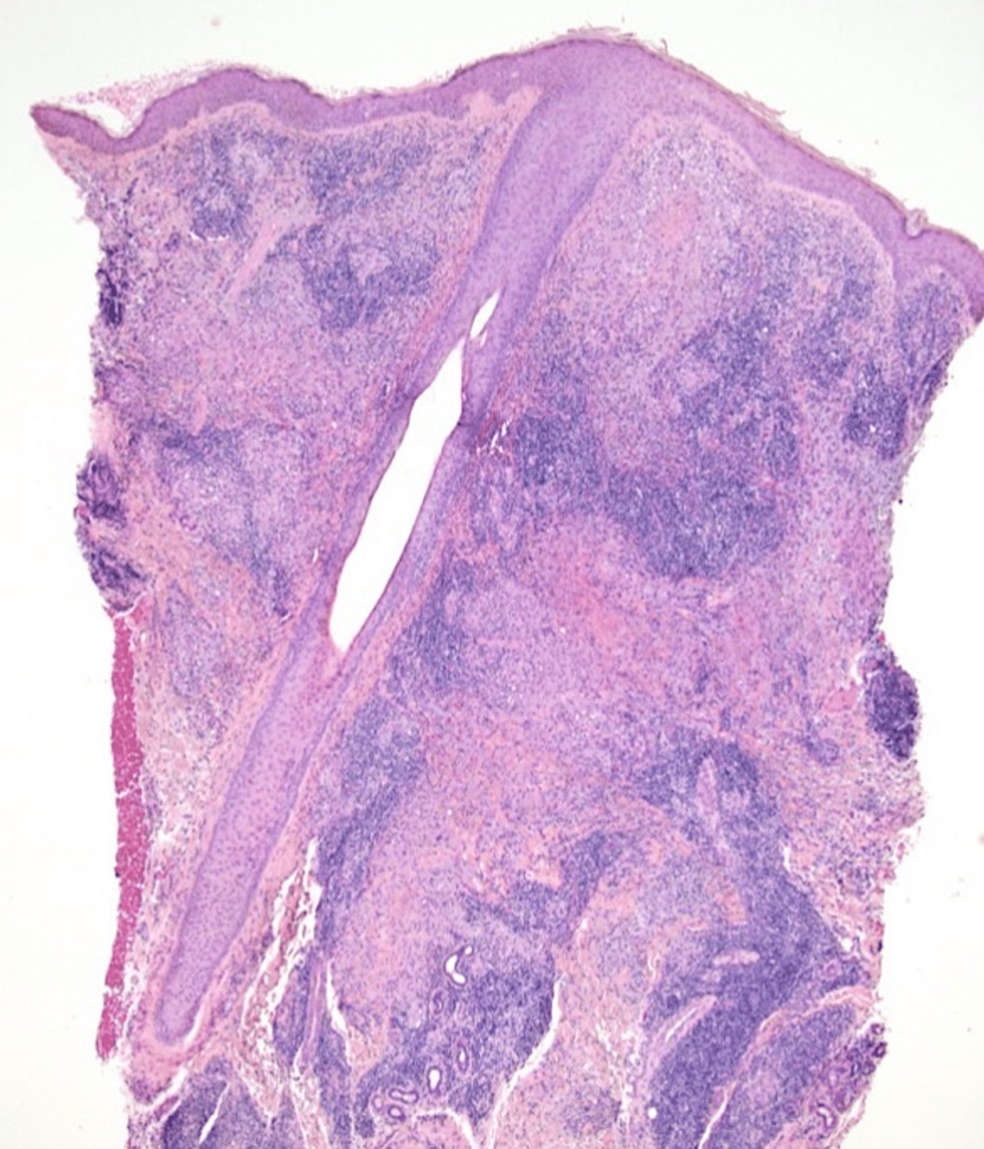
H&E stain of left lateral neck punch biopsy at 10x magnification H&E: Hematoxylin & Eosin

**Figure 3:**
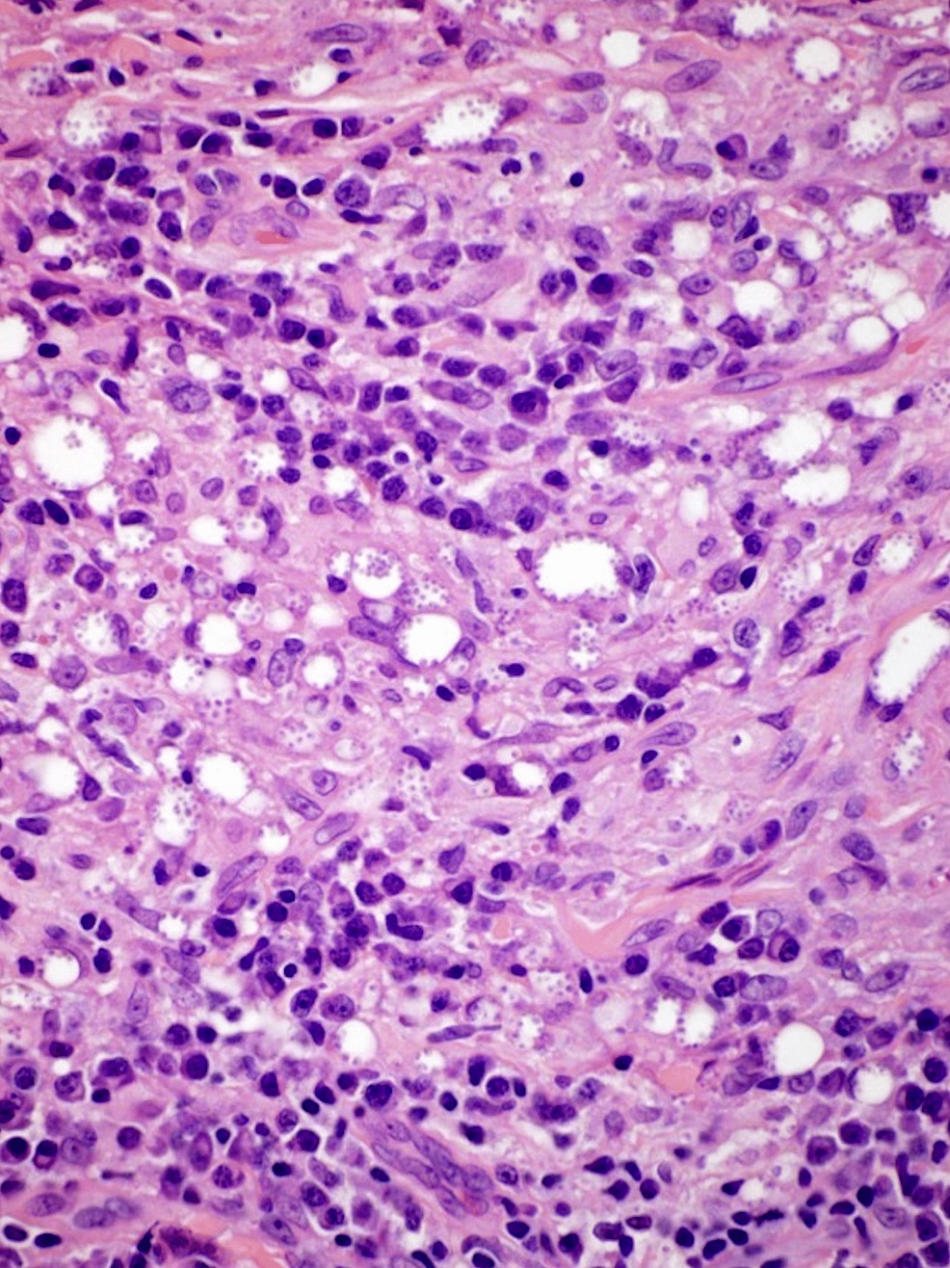
H&E stain of left lateral neck punch biopsy at 100x magnification A.W. Dermatopathology Service Microscopic Description: Sections show skin with a diffuse dermal mixed inflammatory infiltrate composed of lymphocytes, histiocytes, neutrophils, and plasma cells. Necrotic inflammation is seen. There are numerous small vacuolated histiocytes that contain numerous small round organisms with eccentrically located dark bodies. PAS, GMS, and Giemsa stains are carried out with adequate controls. H&E: Hematoxylin & Eosin; PAS: periodic acid-Schiff; GMS: Grocott-Gomori methenamine silver

The patient had recently traveled to Mexico where he developed a "persistent bug bite" with the development of new lesions. The patient did not respond to an initial treatment combination of topical ketoconazole with oral trimethoprim/sulfamethoxazoleand cefalexin. An initial punch biopsy of the left lateral neck lesion was consistent with leishmaniasis. Hematoxylin & Eosin stains of the neck punch biopsy can be appreciated in Figure 2 and Figure 3 revealing "diffuse dermal mixed inflammatory infiltrate composed of lymphocytes, histiocytes, neutrophils and plasma cells. Necrotic granulomatous inflammation is seen. There are numerous small vacuolated histiocytes which contain numerous small organisms with eccentrically located dark bodies."

A shave biopsy of the left posterior ear was performed; pathology results were consistent with leishmaniasis infection. The patient also presented with erythematous papules distributed on the right ventral forearm strongly suspected to be arthropod assault with underlying leishmaniasis infection; a 3.5 mm punch biopsy was performed. Pathology results showed granulomatous and necrotizing dermatitis, highly suspicious for cutaneous leishmaniasis. The pathology report noted that no definitive organisms were present on multiple special stains; however, the tissue reaction was similar to that seen from the posterior ear and was highly suggestive of leishmaniasis infection. Antimonial therapy and microwave therapies were discussed but were not accessible to the patient. The patient was counseled, and a follow-up was recommended to discuss treatment with cryotherapy using liquid nitrogen.

In September 2018, the patient returned for further evaluation, management, and treatment. Cryotherapy was recommended as an alternative treatment for persistent lesions. Lesions located on the left superior anterior neck, left superior posterior ear, and right ventral proximal forearm were treated with freeze-thaw cycles with liquid nitrogen. Multiple freeze-thaw cycles were used on larger lesions during the initial treatment. This procedure was medically necessary because the treated lesions were new, inflamed, intensely itchy, and infectious. The patient's consent was obtained including but not limited to risks of recurrence and incomplete removal of the infection or failure of treatment. The patient followed up every two weeks. During each visit, the areas were assessed, and liquid nitrogen was applied depending on the level of visible improvement. Postoperative care between visits involved daily cleansing and mupirocin ointment to avoid secondary infection and improve healing.

**Figure 4:**
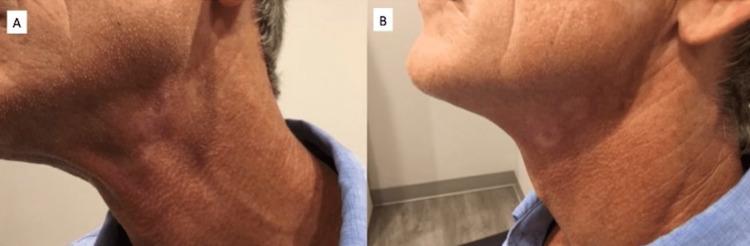
Clinical pictures after cryotherapy Image A (Left): Anterolateral view of neck lesions three months after final cryotherapy treatment. Image B (Right): Lateral view of neck lesions three months after final cryotherapy treatment.

In October 2018, the lesions were improving and were treated again with liquid nitrogen. In November 2018, the lesions treated at the previous visits were almost clear. One lesion on the left posterior ear was treated again with liquid nitrogen. In January 2019, the lesion on the left posterior ear was retreated. In May 2019, the lesions were clear and required no further treatment. No adverse events were noted throughout treatment. The patient was counseled and instructed to return for a follow-up visit in three months. Figure 4 displays the healed neck lesions over three months after the final cryotherapy treatment cycle. The patient remains disease free more than 5 years after initial treatment with no signs of relapse or need for additional treatment.

## Discussion

After multiple sessions of cryotherapy treatments with liquid nitrogen, the nodules on the arm and neck significantly improved. A larger ulcer located on the left ear required two additional treatments spaced six weeks apart before significant improvement. Once all the lesions were visibly healed, the patient followed up every three to six months. At these follow-up visits, the patient had no signs of relapse and had not developed any new lesions. Our patient has had no visible sign of relapse since their last treatment five years ago.

Leishmaniasis is a significant health concern affecting a wide range of populations across the globe. In our practice, we have found that biweekly use of cryotherapy can significantly improve lesions caused by this infection. The logic behind the use of cryotherapy was adapted from the treating physician's knowledge of the organism's sensitivity to temperature changes [10]. While conventional treatments involve high-temperature thermotherapy, it was speculated that the use of low-temperature cryotherapy with liquid nitrogen would exploit the organism's sensitivity to temperature, producing a therapeutic effect.

Our findings are congruent with a recent publication by Mosleh et al. In this clinical study, 120 patients with leishmaniasis were treated with weekly cryotherapy sessions [2]. After one to four sessions, 84% of lesions were cured. The remaining 16% of lesions were cured with an increased number of treatments ranging from 5-7 total sessions. This study found no recurrence during their four-week follow-up period and no relapse in the 78 patients that followed up three years after their last treatment [2]. This clinical study in combination with the results in our practice validates the efficacy of cryotherapy to treat patients with cutaneous leishmaniasis.

Additionally, a meta-analysis published by *BMC Infectious Diseases* in 2016 concluded that the efficacy of cryotherapy is similar to pentavalent antimonials for cutaneous leishmaniasis treatment [6]. The accessibility and minimal side effect profile make liquid nitrogen a favorable treatment compared to pentavalent antimonial therapy. However, when used in combination, the synergistic effects of cryotherapy and other first-line antileishmanial agents may prove favorable in treating the infection. Reducing the parasitic load in the cutaneous lesions with liquid nitrogen could decrease the effective dose of systemic therapy, in turn decreasing the risk of toxic adverse effects. Treating the cutaneous stage early will prevent later visceral presentations of the infection and decrease both disease- and treatment-related mortality. Cryotherapy should be studied alone and in combination with other therapies to standardize a treatment plan that is low-risk and more accessible for patients with leishmaniasis in areas with limited healthcare. Randomized control and comparative studies with larger representative sample populations should be conducted to establish more evidence-based results and standardize cryotherapy as a first-line treatment for cutaneous leishmaniasis. 

## Conclusions

Weekly to bi-weekly intervals of one to four cryotherapy sessions cured lesions caused by leishmaniasis infection, especially smaller lesions. Cryotherapy with liquid nitrogen is a safe, effective, cost-efficient, and accessible treatment modality for cutaneous leishmaniasis, unlike other first-line therapies. For these reasons, cryotherapy should be researched further and adopted as a first-line treatment for cutaneous leishmaniasis, especially in low-resource, developing countries where leishmaniasis is endemic.
